# An Investigation for Skin Tissue Regeneration Enhancement/Augmentation by Curcumin-Loaded Self-Emulsifying Drug Delivery System (SEDDS)

**DOI:** 10.3390/polym14142904

**Published:** 2022-07-17

**Authors:** Saima Mahmood, Prapanna Bhattarai, Nauman Rahim Khan, Zakia Subhan, Ghulam Razaque, Hassan A. Albarqi, Abdulsalam A. Alqahtani, Ali Alasiri, Lin Zhu

**Affiliations:** 1 Department of Pharmaceutics, Faculty of Pharmacy, Gomal University, Dera Ismail Khan 29050, KPK, Pakistan; dr_saimamahmood@hotmail.com; 2 Gomal Centre for Skin/Regenerative Medicine and Drug Delivery Research, Faculty of Pharmacy, Gomal University, Dera Ismail Khan 29050, KPK, Pakistan; 3 Irma Lerma Rangel College of Pharmacy, Texas A&M University, College Station, TX 77843, USA; pbhattarai@tamu.edu; 4 Department of Pharmacy, Kohat University of Science and Technology, Kohat 26000, KPK, Pakistan; 5 Institute of Medical Sciences, Khyber Medical University, Kohat 26000, KPK, Pakistan; zakiabilal10@gmail.com; 6 Faculty of Pharmacy, University of Baluchistan, Quetta 87300, Baluchistan, Pakistan; razaque_pharmacyuob@yahoo.com; 7 Department of Pharmaceutics, College of Pharmacy, Najran University, Najran 55461, Saudi Arabia; haalbarqi@nu.edu.sa (H.A.A.); aamari@nu.edu.sa (A.A.A.); aalasiri@nu.edu.sa (A.A.)

**Keywords:** self-emulsifying drug delivery system (SEDDS), curcumin, nanomedicine, wound healing, skin regeneration, diabetic wound

## Abstract

Diabetes, one of the global metabolic disorders, is often associated with delayed wound healing due to the elevated level of free radicals at the wound site, which hampers skin regeneration. This study aimed at developing a curcumin-loaded self-emulsifying drug delivery system (SEDDS) for diabetic wound healing and skin tissue regeneration. For this purpose, various curcumin-loaded SEDDS formulations were prepared and optimized. Then, the SEDDS formulations were characterized by the emulsion droplet size, surface charge, drug content/entrapment efficiency, drug release, and stability. In vitro, the formulations were assessed for the cellular uptake, cytotoxicity, cell migration, and inhibition of the intracellular ROS production in the NIH3T3 fibroblasts. In vivo, the formulations’ wound healing and skin regeneration potential were evaluated on the induced diabetic rats. The results indicated that, after being dispersed in the aqueous medium, the optimized SEDDS formulation was readily emulsified and formed a homogenous dispersion with a droplet size of 37.29 ± 3.47 nm, surface charge of −20.75 ± 0.07 mV, and PDI value of less than 0.3. The drug content in the optimized formulation was found to be 70.51% ± 2.31%, with an encapsulation efficiency of 87.36% ± 0.61%. The SEDDS showed a delayed drug release pattern compared to the pure drug solution, and the drug release rate followed the Fickian diffusion kinetically. In the cell culture, the formulations showed lower cytotoxicity, higher cellular uptake, and increased ROS production inhibition, and promoted the cell migration in the scratch assay compared to the pure drug. The in vivo data indicated that the curcumin-loaded SEDDS-treated diabetic rats had significantly faster-wound healing and re-epithelialization compared with the untreated and pure drug-treated groups. Our findings in this work suggest that the curcumin-loaded SEDDS might have great potential in facilitating diabetic wound healing and skin tissue regeneration.

## 1. Introduction

Diabetes mellitus is a diverse metabolic disease described by hyperglycemia due to a relative or absolute lack of insulin [1]. Generally, diabetes mellitus is classified into two types, including type 1 diabetes and type 2 diabetes, with a considerable difference between the two types [2]. Type 1 diabetes, which is most common in children and young adults, is an autoimmune disease, which leads to the destruction of the insulin-producing pancreatic beta cells in the islets of Langerhans, which results in interference in insulin secretion [1]. 

Type 2 diabetes is related to insulin resistance, although the level of insulin is high [2]. It is a considerable health burden with growing prevalence globally. In 2021, around 537 million people were affected with diabetes with an estimated 6.7 million deaths. The number of diabetics is predicted to rise to 643 million by the year 2030 and 783 million by the year 2045 [3]. Treatment options for type 2 diabetes mellitus consist of alpha-glucosidase inhibitors, insulin sensitizers, secretagogues, and insulin, while for diabetic wounds, treatment includes the use of topical and/or oral antibiotics and controlling the blood glucose level with insulin or other hypoglycemic drugs [4].

Diabetes is often accompanied by secondary pathological conditions, such as retinopathy, nephropathy, and neuropathy. Diabetic patients also suffer from chronic wounds [5], which may require amputation of the affected body parts if not intervened in time [6,7]. The wounds in diabetes are characterized by high oxidative stress [8], lack of tissue renewal, impaired wound healing, and, most importantly, secondary infections [9,10].

Curcumin (1,7-bis(4-hydroxy-3-methoxyphenyl)-1,6-heptadiene-3,5-dione), the most active polyphenolic component of turmeric compounds, is acquired from the dried rhizome of *Curcuma longa* [11,12]. Curcumin displays numerous properties, including antioxidant, anti-inflammatory [13], anti-microbial [14], anti-cancer, anti-mutagenic [15], and antidiabetic effects [16,17].

Curcumin has also attracted scientific interest as a prospective therapeutic agent in treating and managing diabetic complications [18]. It was reported that curcumin can enhance the biosynthesis of extracellular matrix (ECM) and influence the transforming growth factor-beta (TGF-β) pathway in wounds, which translates into wound contraction [19]. Curcumin can also inhibit DNA breakage and lipid peroxidation and reduces inflammation via interfering with the nuclear factor kappa B signaling pathway [20,21]. Moreover, curcumin encourages a rapid shift from the inflammatory phase to the proliferative phase of wound healing, resulting in increased neovascularization, high collagen deposition, rapid re-epithelialization, and tissue formation [21]. 

In diabetes, persistent hyperglycemia enhances protein glycation, the auto-oxidation of glucose, and polyol metabolism, resulting in the overproduction of reactive oxygen species (ROS). This, in turn, enhances the oxidative chemical transformation of proteins, lipids, and DNA in various tissues [22]. It is well-known that curcumin is a potent antioxidant that can effectively inhibit ROS production in tissues [23,24]. The administration of curcumin during diabetes mellitus ameliorates oxidative damage and apoptosis in cardiomyocytes by inducing PI3K/Akt and SIRT1/FOX1 pathways [25].

A lecithinized curcumin-containing delivery system (Meriva^®^) was employed for managing diabetic retinopathy and microangiopathy with proven significant results [26]. In another study, curcumin and quercetin were used in combination for the treatment of type 2 diabetes mellitus in streptozotocin-induced rats [16]. Similarly, the coadministration of polypeptide-k and curcumin was used for the treatment of diabetes mellitus [27].

However, due to the unfavorable physicochemical properties, such as poor water solubility, high hydrophobicity, photosensitivity, and low stability, the use of curcumin in diabetic wound management is limited [28]. SEDDS is considered an alternative low-cost approach to formulate hydrophobic drugs for treating open incision wounds.

A self-emulsifying drug delivery system (SEDDS) is an effective and less expensive delivery system for hydrophobic drugs as compared to other nanocarriers, such as liposomes and nanoparticles [29,30]. The SEDDS formulations are usually isotropic mixtures of oils, suitable stabilizing surfactants, and co-surfactants that emulsify spontaneously upon mild agitation in an aqueous media and result in the development of nano-sized (<200 nm droplet size) oil-in-water emulsions [15,31,32,33]. 

The SEDDS formulations can improve drug bioavailability via various mechanisms, including drug solubilization, droplet size reduction, better membrane permeability, and the protection of drugs against chemical/enzymatic degradation [34,35]. Different SEDDS formulations were developed and tested with the perspective of skin applications, such as the *Piper cubeba* essential oil SEDDS for wound healing applications [36], *Opuntia ficus-indica* fixed oil SEDDS for wound healing purposes [37], curcumin SEDDS for transdermal drug delivery [38] and SEDDS for the topical delivery of mangosteen peel (*Garcinia mangostana* L.) [39].

This study aimed at developing, optimizing, and evaluating curcumin-loaded SEDDS formulations as a novel nano carrier-based wound healing platform. The curcumin SEDDS are envisioned to exhibit lower cytotoxicity and higher cellular uptake, promote rapid cell migration, inhibit excessive ROS production, and hasten skin regeneration compared to pure curcumin.

## 2. Materials and Methods

### 2.1. Materials

Curcumin, polysorbate 80 (tween-80), Vitamin E Succinate, disodium hydrogen orthophosphate, sodium chloride, monobasic potassium phosphate, dimethyl sulfoxide (DMSO), 3-(4,5-Dimethylthiazol-2-yl)-2,5-diphenyltetrazolium bromide (MTT), 2′,7′-dichlorodihydrofluorescein diacetate (H2DCFDA), and Triton X-100 were purchased from Sigma-Aldrich (St. Louis, MO, USA). PEG-600 was purchased from Beantown chemicals (BTC, Hudson, NH, USA), ethyl oleate from Acros organics (Geel, Belgium), hydrochloric acid from Merck Germany, and cellulose dialysis membrane from Spectrum Labs VWR Scientific (Radnor, PA, USA). The Dulbecco’s modified Eagle’s medium (DMEM), penicillin−streptomycin (PS) solution 100×, and trypsin−ethylenediaminetetraacetic acid were purchased from Invitrogen Corp. (Carlsbad, CA, USA). Fetal bovine serum (FBS) and Hank’s balanced salt solution (HBSS) were purchased from Mediatech (Manassas, VA, USA), and streptozotocin (STZ) was purchased from AG Scientific (San Diego, CA, USA). All chemicals were used without any further purification.

### 2.2. Preparation and Optimization of Curcumin-Loaded SEDDS

The curcumin-loaded SEDDS formulation contains ethyl oleate (oil), tween 80 (surfactant), PEG-600 (co-surfactant), and curcumin. A total of twenty-two SEDDS formulations were prepared and optimized by varying the contents of the inactive ingredients within their solubility/miscibility limits, using a previously reported method [40]. They were then analyzed visually for their stability and homogeneity, indicated by the absence of creaming, phase separation, flocculation, and sedimentation. 

Briefly, the curcumin-loaded SEDDS were prepared by dissolving the curcumin in PEG-600, followed by the addition of tween 80 under continuous stirring at 400 revolutions per minute. The ethyl oleate was then added to the mixture and stirred for 15 min to form a homogeneous oil phase (internal phase). The pre-concentrate SEDDS (i.e., oil phase) was then added dropwise into the water phase (external phase) under stirring for 10 min to form a homogenized, transparent O/W nanoemulsion. The SEDDS formulations developed are listed in Table 1.

### 2.3. Droplet Size, Zeta Potential, and PDI

The droplet size, zeta potential, and poly dispersibility index (PDI) of the SEDDS formulations were analyzed by dynamic light scattering technique (Nanobrook 90Plus Zeta PALS, Zeta Potential analyzer, Brookhaven Instruments Corporation, Holtsville, NY, USA) at 25 °C with a scattering angle of 90°. All samples were diluted ten times with pH 7.4 PBS before analysis. Each formulation was analyzed in triplicate.

### 2.4. Drug Content (DC) and Entrapment Efficiency (EE)

One milliliter of each formulation was taken in Eppendorf tubes, treated with 1 mL of dimethyl sulphoxide (DMSO), and centrifuged (Eppendorf centrifuge:5810 R-15 Amp version, Germany) at 4 °C for 30 min at 4000 rpm. After centrifugation, the supernatant was carefully decanted off from the lower layer. Each layer’s fluorescence intensity was determined at an excitation wavelength of 440 nm and emission wavelength of 550 nm using a microplate reader (Tecan microplate reader, Gordig, Austria). The fluorescence intensity (FI) of the formulation (without centrifugation/phase separation) was measured to approximate the total drug in the formulation. The entrapment efficiency and drug content were calculated using the following relations.

(1) $$\mathrm{DC}=\frac{\mathrm{FI}\;\mathrm{of}\;\mathrm{oil}\;\mathrm{phase}+\mathrm{FI}\;\mathrm{of}\;\mathrm{aqueous}\;\mathrm{phase}}{\mathrm{FI}\;\mathrm{of}\;\mathrm{uncentrifuged}\;\mathrm{formulation}}\times 100\%$$

(2) $$\mathrm{EE}=\frac{\mathrm{FI}\;\mathrm{of}\;\mathrm{whole}\;\mathrm{formulation}-\mathrm{FI}\;\mathrm{of}\;\mathrm{oil}\;\mathrm{phase}}{\mathrm{FI}\;\mathrm{of}\;\mathrm{whole}\;(\mathrm{uncentrifuged}\;\mathrm{formulation})}\times 100\%$$

### 2.5. In Vitro Drug Release

The in vitro drug release of the SEDDS formulations was performed using a previously reported (dialysis bag) method [41,42,43]. Briefly, the formulation (1 mL) was dialyzed (in dialysis tubing; molecular weight cutoff 12,000 to 14,000 Da) against 20 mL of PBS (pH 7.4 with added 0.1% tween 80 to simulate the “sink” condition) under stirring at 200 rpm and maintained at 37 ± 2 °C. The experimental setup was protected from the light because of curcumin’s photosensitivity. 

A 0.5 mL aliquot of the dialysate was withdrawn at 0, 2, 4, 6, 8,10, 12, 24, and 48 h, respectively, and replaced with an equal volume of the fresh buffer. The released curcumin in the collected samples was analyzed on a microplate reader (Tecan, USA) at an excitation wavelength of 440 nm and an emission wavelength of 550 nm. The experiment was repeated three times. The pure drug release profile was also checked the same way, for which drug quantity equivalent to that available in 1 mL of the formulation was pre-dissolved in DMSO and PBS pH 7.4.

The drug release data were fitted into the Korsmeyyer Peppas equation as given below to determine the mechanism of drug release.

(3) $$\frac{\mathrm{Mt}}{M\infty }={\mathrm{Kt}}^{n}$$

where Mt/M∞ is a fraction of the drug released at time t, K is the release rate constant, and n is the release exponent. The 0.45 ≤ n corresponds to a Fickian diffusion mechanism, 0.45 < n < 0.89 to non-Fickian transport, n = 0.89 to Case II (relaxational) transport, and n > 0.89 to super case II transport [44].

### 2.6. Stability of the Nano-Emulsion Formed by SEDDS

The effects of the dilution and buffer pH on the stability of the nano-emulsion formed by SEDDS were studied as a function of their droplet size and PDI. Briefly, 200, 100, 40, 20, 10, 6.67, 5, 3.33, and 2.5 µL of the SEDDS formulations were diluted to 1 mL (equal to 5, 10, 25, 50, 100, 150, 200, 300, and 400 fold dilutions, correspondingly) with PBS at pH 5.5, 6.5, 7.4, or 8.5. Then, the nano-emulsions droplet size, PDI, and zeta potential were measured on a Nanobrook zeta sizer (BrookHavens Instruments Corporation, Holtsville, NY, USA). The nano-emulsions were also observed for any physical changes, i.e., coalescence and/or precipitation.

### 2.7. Thermodynamic Stability of SEDDS

Although the nano-emulsions have been reported to be kinetically stable, the nano-emulsion “thermodynamic” stability needs to be evaluated to exclude metastable and/or unstable formulations under various circumstances. The SEDDS formulations’ thermodynamic stability was assessed by the following tests [45]. Each test was performed in triplicates.

#### 2.7.1. Centrifugation

Three milliliters of the SEDDS formulations were centrifuged at 3500 rpm for 30 min. They were observed for turbidity, phase separation, and (or) cracking.

#### 2.7.2. Heating/Cooling Cycle

The thermal stability of the SEDDS formulations was estimated by subjecting the formulation to a heat/cool cycle. Briefly, the formulation was cooled to 4 °C in a refrigerator for 48 h, followed by heating in an incubator at 45 °C for 48 h.

#### 2.7.3. Freeze–Thaw Cycle

The SEDDS formulations were stored at −20 °C for 24 h and then placed at room temperature until they thawed. The visual characteristics of the formulation were checked after returning to their original liquid state at room temperature. The formulation was considered thermodynamically stable if it could readily return to the original liquid state without any noticeable phase separation, aggregation, or precipitation.

### 2.8. Long-Term Storage Stability

The SEDDS formulations were stored under standard conditions (25 ± 2 °C, RH 65 ± 5%) [27] for 60 days, followed by droplet size measurement. In addition, the drug content (fluorescence) in the formulations was measured by a microplate reader (Tecan, Gordig, Austria) after storing them for five months under the standard conditions mentioned above.

### 2.9. Cytotoxicity/Cell Viability

The cytotoxicity of the SEDDS formulations was evaluated on the NIH3T3 fibroblasts using a previously reported method [46]. Briefly, the NIH3T3 cells were cultured in the DMEM supplemented with 10% FBS and 1% PS at 37 °C under 5% CO_2_. The cells were seeded at a density of 5 × 10^3^ cells/well in 96-well plates 24 h before treatments. The cells were exposed to the treatments in the curcumin concentration range of 1.95 µg/mL to 4000 µg/mL for 48 h at 37 °C. After 48 h, the medium was removed and replaced by 180 µL of fresh medium and 20 µL of MTT reagent (5 mg/mL), followed by incubation at 37 °C for 4 h. Then, the culture medium was aspirated out, and 200 µL/well DMSO was added to the cells. The absorbance was recorded at 570 nm on a microplate reader. The cell viability was then calculated using the following equation.

(4) $$\mathrm{Cell}\;\mathrm{viability}\;=\frac{A1}{A2}\times 100\%$$

where A1 is the absorbance of treated cells and A2 is the absorbance of untreated cells. The results are presented as the mean of six experiments.

### 2.10. Cell Migration/Scratch Assay

The effect of the SEDDS formulations on cell migration was evaluated using the scratch assay. Briefly, the NIH3T3 cells were seeded in a 6-well plate at a cell density of 1 × 10^5^ cells/well. On the next day, a scratch was made on the adhered cells with a 200 µL sterile micropipette tip, and the cells were washed gently by serum-free medium. The cells were treated with the formulations and/or pure drug solution at 10 µM curcumin concentration. The scratch images were taken at 0, 24, and 48 h by a Nikon Eclipse Ti fluorescence microscope at 40X magnification. The width of the scratch was measured.

### 2.11. Intracellular ROS Measurement/Antioxidant Assay

The intracellular ROS level was measured to evaluate the antioxidant potential of the curcumin-loaded SEDDS by a previously reported method [43]. Briefly, the NIH3T3 cells were seeded in a 24 well plate at 5 × 10^4^ cells/well and incubated for 24 h. The next day, the ROS production was induced by incubating the cells with 60 µM vitamin E succinate for 24 h. The ROS-induced cells were treated with the pure drug or SEDDS formulations at 4 µg/mL curcumin for an additional 24 h. 

To determine the intracellular ROS, the treated cells were gently washed twice with PBS, followed by incubating with 10 µM H_2_DCFDA in the DMEM for 30 min. Images of the ROS-producing cells were taken with a Nikon Eclipse Ti fluorescence microscope. The cells were then lysed by 2% Triton X-100 and centrifuged at 1000 rpm for 10 min. The fluorescence intensity of the supernatant was measured by a microplate reader at an excitation wavelength of 485 nm and an emission wavelength of 530 nm. The fluorescence intensity of the cells treated only with vitamin E succinate was taken as 100%.

### 2.12. Cellular Uptake

The NIH3T3 cells were seeded into the 24-well plates at 1 × 10^5^ cells/well density and incubated overnight. The cells were treated with the SEDDS formulations or pure drug at low (10 µg/mL) and high curcumin doses (100 µg/mL) for 1 or 4 h, followed by observation under a Nikon Eclipse Ti fluorescence microscope. Then, the cells were trypsinized and centrifuged at 1000 rpm for 2 min. The cell pellets were re-suspended in serum-free DMEM and analyzed for fluorescence-activated cell sorting (FACS) using a BD Accuri C6 Plus Flow cytometer. The cells were gated upon acquisition using forward versus side scatter to exclude cell debris and dead cells. The untreated cells served as the negative control. The cells (2 × 10^4^ cell counts/events) were recorded and analyzed with BD Accuri C6 Plus Software.

### 2.13. Diabetic Wound Healing

Healthy male *Sprague Dawley rats*, weighing 200 to 250 g were purchased and acclimatized for 14 days with easy access to food and water at 19–23 °C with a 12 h light–dark cycle. Before the diabetes induction, the rats were kept fasting for 24 h with easy access to water. They were weighed, and their fasting blood glucose levels were determined using a glucometer (CodeFree, SD Biosensor, Korea). The rats were injected with a single dose of freshly prepared streptozotocin solution intraperitoneally at a dose of 50 mg/kg body weight of the animals. The blood glucose levels of the animals were monitored starting on day 3 of the streptozotocin injection, and the rats were considered diabetic when their blood glucose levels were >250 mg/dL [47]. 

Following the diabetes induction, the diabetic rats were randomly divided into three groups (n = 8 for each group), i.e., untreated, SEDDS, and pure drug groups. The rats were anesthetized by intraperitoneal injection of the ketamine (100 mg/kg), and xylazine (10 mg/kg) mixture, and the back hair of the rats was shaved. An open incision wound was inflicted on the mid-dorsal thoracic region of the rat with the help of sterilized forceps and surgical scissors. Following the wound infliction, the treatments were applied to the injured area, covered with sterile gauze, and adhered with 3M adhesive tape. 

The untreated group received only the gauze application, the SEDDS group received the optimized formulation (4 mg/mL) application, and the pure drug group received curcumin solution (4 mg/mL in a mixture of DMSO and PBS pH 7.4). The treatments were applied daily until complete wound healing was observed. The photographs of wounds were taken by a Canon D5200 camera (Japan) on days 0, 3, 7, 14, and 16 post-administration. The wound size was analyzed by Image J software (version.1.53k. US National Institutes of Health, Bethesda, MD, USA). The percent re-epithelialization (RE) was then calculated using the following relation [48].

(5) $$\mathrm{RE}\;=\frac{\left(\mathrm{Wound}\;\mathrm{size}\;\mathrm{at}\;t=0\right)-\left(\mathrm{Wound}\;\mathrm{size}\;\mathrm{at}\;t\right)}{\mathrm{Wound}\;\mathrm{size}\;\mathrm{at}\;t=0}\times 100\%$$

The institutional Ethical Review Board approved the animal study protocol vide reference no. 502/QEC/GU, dated 29 March 2019, Gomal University Pakistan.

### 2.14. Statistical Analysis

The data are expressed as the mean ± standard deviation. Student’s *t*-test or analysis of variance (ANOVA) followed by post hoc analysis was used with the significance level set at *p* < 0.05.

## 3. Results and Discussion

### 3.1. Formulation Optimization

For formulation optimization, a grading system was used as previously reported [49,50,51]. The formulations were observed for 15 days upon the preparation. The optical observation, physical appearance, and emulsification time of the formulations are shown in Table 1. The results showed that only the formulations F1, F2, and F3 met the Grade A criteria, i.e., quick emulsification and the absence of phase separation and drug precipitation. Formulations F4 to F10 and F12 to F19 underwent phase separation without drug precipitation and thus were graded B. It took a long time for formulations F11 and F20 to F22 to emulsify. In addition, phase separation and drug precipitation were observed in these formulations, which were assigned Grade C.

### 3.2. Droplet Size, Zeta Potential, and PDI

Upon mild agitation, the internal phase of the formulations F1, F2, and F3 was rapidly and homogeneously dispersed in the continuous phase to form a nano-emulsion with the PDI values in the range of 0.258 ± 0.015 to 0.328 ± 0.027. Among them, the smallest droplet size was observed for the F3 formulation (37.29 ± 3.47 nm, Student’s *t*-test, *p* < 0.05). The zeta potential results indicated that all three formulations bore a negative charge in the range of −13.22 ± 0.51 mV to −20.75 ± 0.07 mV, where the zeta potential of the F3 formulation was much higher than the other formulations. 

**Figure 1:**
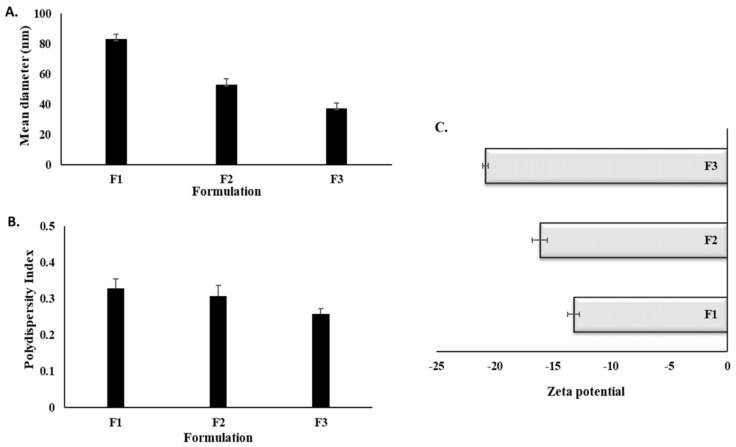
Droplet size (**A**), PDI (**B**), and zeta potential, and (**C**) results of the optimized SEDDS.

The droplet size results, zeta potential, and PDI are depicted in Figure 1. With decreasing oil phase and increasing surfactant concentration, a significant size reduction was observed for SEDDS, where a smaller droplet size is envisioned to ensure colloidal stability owing to the higher surface area aiding in drug absorption [52,53]. Furthermore, small droplet size emulsions have low residual oxygen content and, thus, also favor the oxidative stability of emulsions [54]. 

Owing to these properties, it was expected that the SEDDS with smaller droplet sizes would provide decent stability to compounds, such as curcumin. In terms of the zeta potential, a significantly higher value was observed for the F3 formulation (Student’s *t*-test, *p* < 0.05) in comparison to other formulations. The negative zeta potential could be attributed to the ionic impurities, free fatty acids, and fatty acid esters in ethyl oleate (internal phase ingredient) or to the adsorption of OH^−^ at the oil–water interface [55,56]. Higher zeta potential values also help ensure colloidal stability due to the electrostatic repulsion of individual droplets [57].

### 3.3. Drug Content and Entrapment Efficiency

The drug contents in formulations F1–F3 were found to be 62.5% ± 2.17% to 70.5% ± 2.31%, and F3 had the highest value. The SEDDS formulations showed the increasing entrapment efficiency from F1 to F3, where all formulations could entrap more than 70% curcumin. The entrapment efficiency of the F3 formulation was 87.5%, which was significantly higher than F1 and F2 (Student’s *t*-test, *p* < 0.05).

### 3.4. In Vitro Drug Release

**Figure 2:**
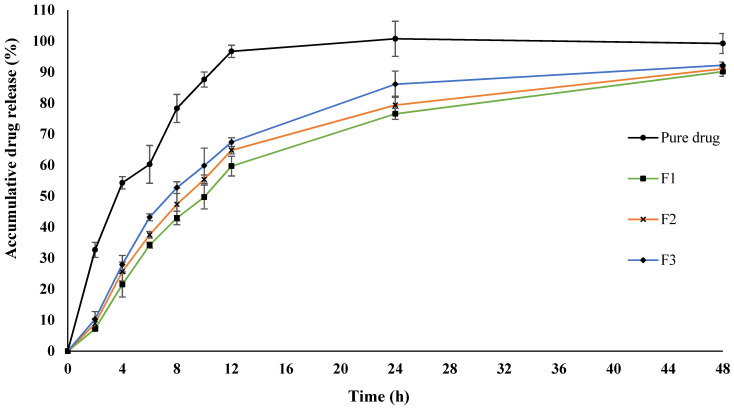
The in vitro drug release profile of curcumin-loaded SEDDS formulations.

Curcumin is a highly hydrophobic drug. Under the simulated sink condition, the pure drug showed a burst release pattern, reaching a plateau (100%) within 12 h. In contrast, the SEDDS formulations showed a sustained release pattern and took more than 48 h to reach the plateau, which was significantly different compared to the free drug solution (ANOVA, *p* < 0.05). The drug release profiles of the pure drug and optimized SEDDS formulations are shown in Figure 2. 

The slow and gradual release of curcumin from SEDDS is favorable for wound healing owing to the prolonged drug action and reduced dosing frequency. Pure drug solution devoid of any external barrier is frequently available for the diffusion, while encapsulated drug in a nanocarrier confers additional covering as a barrier formed by surfactants/co-surfactants/oil phase, thereby, delaying the rapid release and subsequent diffusion across the dialysis membrane [58]. This could be the reason the SEDDS took a longer time to reach a plateau in comparison to the pure drug solution.

By fitting the drug release data into the Korsmeyyer Peppas equation, the results indicated that the F2 and F3 formulations followed the Fickian diffusion mechanism (n = 0.448 for F2 and 0.419 for F3). In contrast, the F1 formulation followed a non-Fickian transport mechanism (n = 0.490) for drug release. The pure drug release profile was found to have an “n” value of 0.257, suggesting a quasi-Fickian diffusion mechanism of drug release.

### 3.5. Stability of the SEDDS and Their Nano-Emulsions

**Figure 3:**
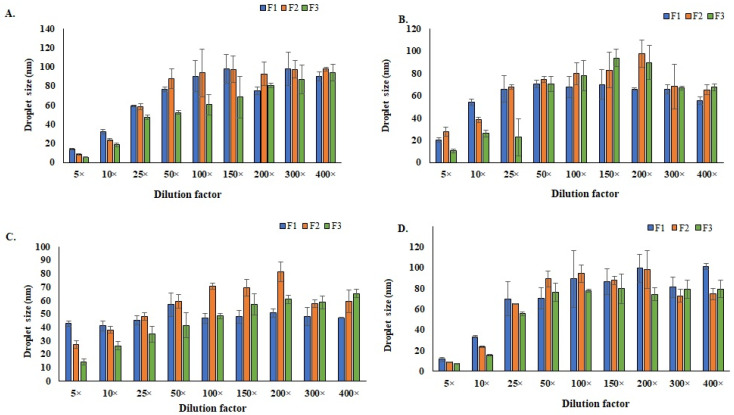
Effects of the dilution and buffer pH on the droplet size of SEDDS (**A**) pH 5.5, (**B**) pH 6.5, (**C**) pH 7.4, and (**D**) pH 8.5.

**Figure 4:**
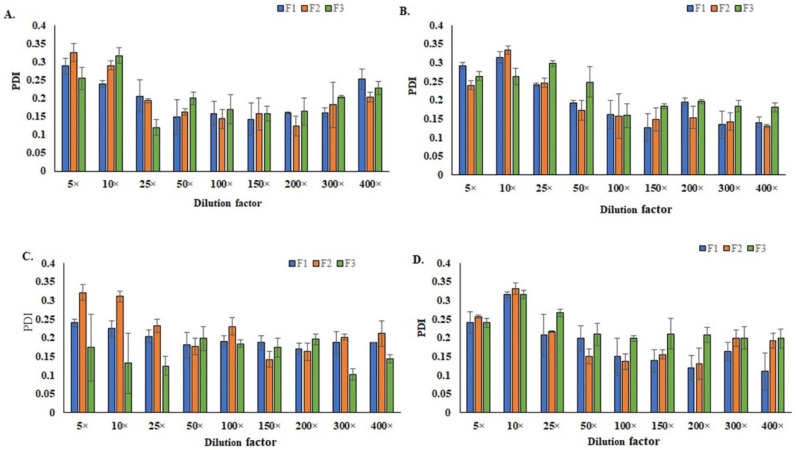
Effects of the dilution and buffer pH on the PDI of SEDDS (**A**) pH 5.5, (**B**) pH 6.5, (**C**) pH 7.4, and (**D**) pH 8.5.

The effects of the dilution and buffer’s pH on the stability of the SEDDS formulations are shown in Figure 3 and Figure 4. With the increase in the dilution folds, the droplet size of the formulations was found to increase, while the PDI was reduced. The effect of the dilution on zeta potential was negligible. The increase in droplet size following dilution could be explained by the progressive dehydration of the non-ionic surfactant head groups causing the packing parameter of the surfactant to tend towards unity (*p* ≈ 1). As a result, there was decreased interfacial tension and increased interfacial flexibility, which is envisaged to promote droplet coalescence [59]. 

However, the particle sizes of all diluted formulations were <120 nm. This could be because the SEDDS formulation consisted of relatively high contents of surfactant and co-surfactant, which stabilized the droplet of nano-emulsions [49]. The change in the buffer’s pH did not significantly affect the droplet sizes of the SEDDS-formed nano-emulsions. In addition, the SEDDS formulations were free from any visible signs of instability when they were subjected to stress conditions, including centrifugation, heating/cooling, freezing/thawing, and large dilutions. The results of the thermodynamic stability tests are depicted in Table 2.

**Figure 5:**
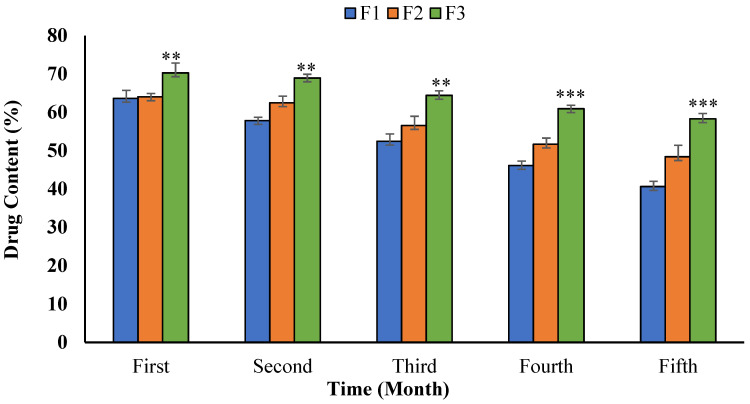
Drug contents of the curcumin-loaded SEDDS over the long-term storage (*** *p* < 0.001, and ** *p* < 0.05) compared to F1, F2, and F3 on day 0.

The long-term stability study of the SEDDS formulations showed an increase in their droplet sizes; however, they were still in the nano range. The droplet size of F1 was found to increase from 83.1 ± 4.52 nm to 113 ± 3.65 nm, while the F2 and F3 sizes were found to increase from 53.5 ± 4.0 nm and 37.29 ± 3.47 nm to 76.0 ± 4.23 nm and 48.5 ± 2.97 nm, respectively. The formulations were stable for 4 months (drug content ≈ 70%, Figure 5) under the standard storage conditions, where the drug content of F3 emulsion was significantly higher than the rest of the formulations (*p* < 0.05). Among all the optimized formulations, the F3 was found to be the best, considering its superior thermodynamic and long-term stability.

### 3.6. Cytotoxicity/Cell Viability

**Figure 6:**
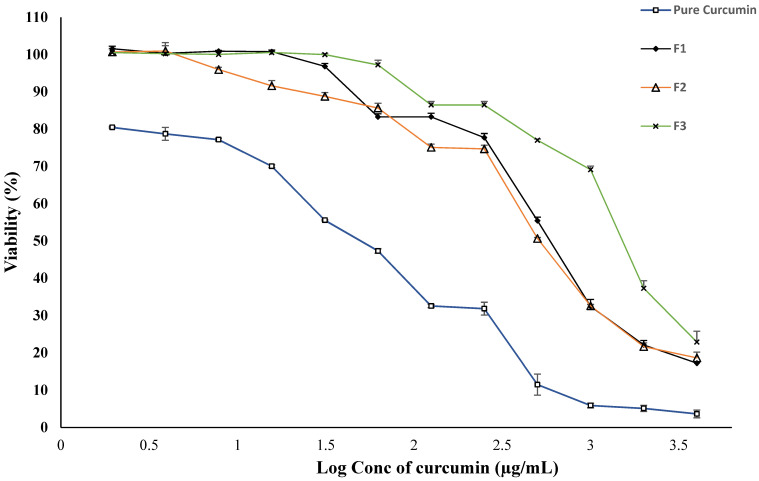
The cell viability results of the curcumin-loaded SEDDS in NIH3T3 cells.

The cytotoxicity of curcumin was reported during its applications [60,61,62], particularly at high concentrations. For treating wound healing, it is of pivotal importance to assess the cytotoxicity of the curcumin-loaded SEDDS formulations to ensure product safety during in vivo applications [62]. The cell viability results of the SEDDS formulations are shown in Figure 6. The pure drug showed significant cytotoxicity even at low concentrations, while after loading to the SEDDS formulations, the curcumin’s cytotoxicity was remarkably decreased. Among the SEDDS formulations, the F3 showed the lowest toxicity. 

The respective blank formulations were also tested for their possible cytotoxicity in NIH3T3 cells, and none of them affected the cell viability confirming their safety upon application (data not presented). Despite its extensive therapeutic applications, pure curcumin has been widely shown to demonstrate non-minimal cytotoxicity at high curcumin concentrations [63]. The low cytotoxicity of curcumin SEDDS compared to pure curcumin in our case could be attributed to the cyto-safe nano-emulsion system developed, making SEDDS less vulnerable to exert cytotoxicity. Curcumin in pure and non-carrier-based delivery systems poses the danger of exerting cytotoxicity by encouraging apoptosis in the cells [47,64], which was significantly reduced when curcumin was formulated into SEDDS.

The cytotoxicity assay showed that curcumin-free SNEDDS were compatible with fibroblast cells with minimal cytotoxic effects only at higher excipient concentrations, signifying that they would not hinder cell proliferation during wound healing [14]. Our results establish the capability of SEDDS to be used for the safe delivery of drugs to cells while evading the cytotoxicity incurred by pure curcumin.

Based on the results and its superiority over other formulations in the stability studies, the F3 formulation was selected for further biological evaluation.

### 3.7. Cell Migration Analysis

**Figure 7:**
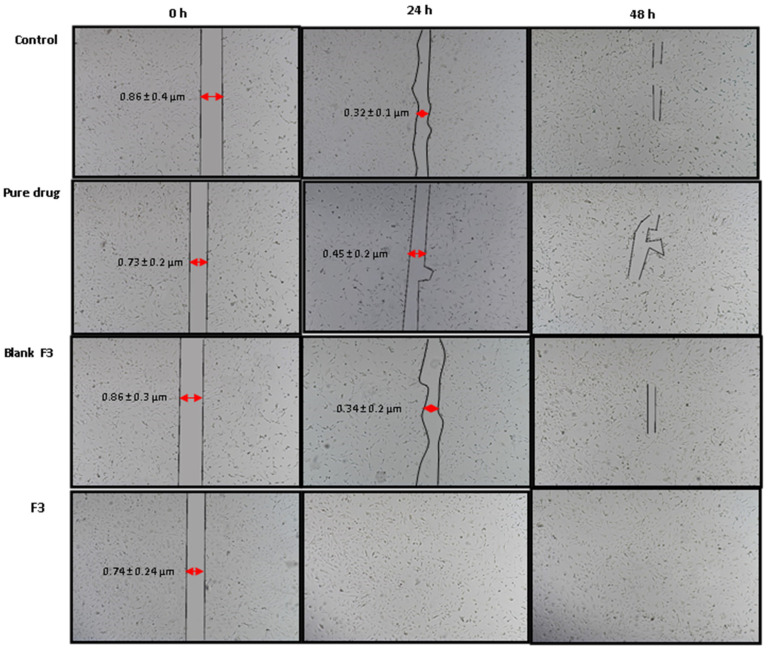
Cell migration upon the curcumin-loaded SEDDS treatment.

The cell migration results are shown in Figure 7. Within 24 h of treatments, the F3 formulation exerted the fastest scratch filling effect among the treatments, while the pure drug, blank formulation, and untreated cells could not fill the scratch even in 48 h. The recruitment of fibroblasts in response to the chemical mediators released at the site of injury as the proinflammatory cytokines and growth factors are crucial in promoting wound healing [65]. Curcumin is a potent antioxidant, antimicrobial, hypoglycemic, and anti-inflammatory drug [66,67]. In this experiment, the SEDDS formulation facilitated drug delivery in the NIH3T3 fibroblasts, which improved curcumin’s anti-inflammatory effects and promoted the “healing” of the in vitro “wound” (scratch).

It is known that the fibroblasts are intimately linked to the ECM and to a large extent, are responsible for the synthesis of the fibrillar constituents of the stroma. In addition, the fibroblasts regulate the function of adjacent epithelial cells through bidirectional interactions and the secretion of growth factors and cytokines [68]. Skin regeneration and wound healing in the diabetic wound is a considerable challenge, which is often compromised due to microbial infiltration into the wound site, reduced migration and proliferation of fibroblasts, decreased angiogenesis, and collagen formation caused by the long-term hyperglycemia [69]. Curcumin has been reported to trigger phosphorylation of c-Src to regulate the protein kinase C (PKC) activation and the phosphorylation of ERK to activate the NF-kB pathway and thus promote cell motility, which contributes to skin tissue regeneration [70].

### 3.8. Intracellular ROS Measurement/Antioxidant Assay

**Figure 8:**
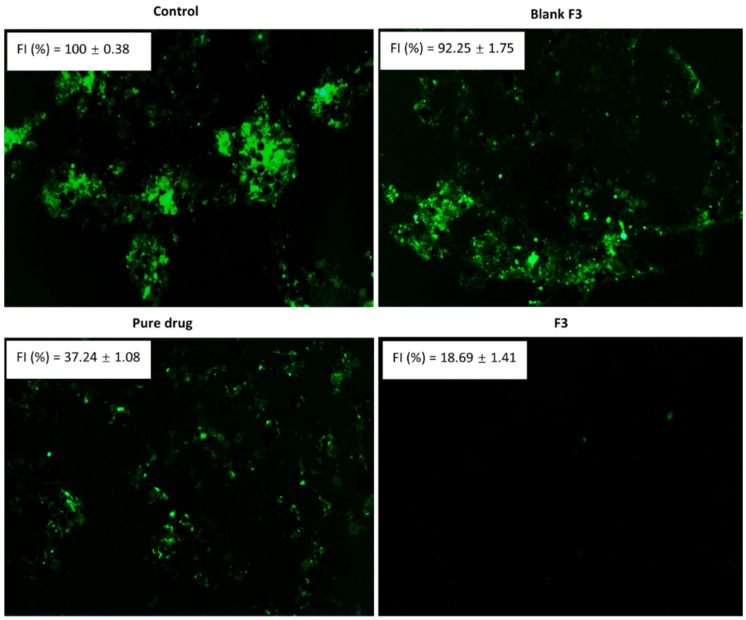
The intracellular ROS level determined by H_2_DCFDA in NIH3T3 cells. (FI = fluorescence intensity).

The intracellular ROS levels in the NIH3T3 cells were determined by the H_2_DCFDA method [43]. The results of fluorescence microscopy (Figure 8) indicated that the Vitamin E Succinate induced a significant ROS production. The pure drug and curcumin-loaded SEDDS showed strong antioxidant effects compared to the blank formulation. The SEDDS formulation significantly increased the antioxidant effect of the loaded curcumin. The microscopy data was consistent with the fluorescence intensity data. Diabetic wounds are often complicated by ROS overproduction at the wound site, which is also a major cause of cell damage in chronic diseases [21,45,71]. 

The excessive ROS production in diabetes is also reported as the result of ferroptosis, a newly discovered form of cell death characterized by the iron-dependent accumulation of lipid peroxides in the pathogenesis of diabetic wound healing [72]. Reducing oxidative stress at the wound site in diabetes is critical to skin regeneration [73]. The free radical scavenging ability of curcumin is thought to arise from its phenolic OH or the CH_2_ moieties of the β-diketone [74]. The curcumin-loaded SEDDS efficiently inhibited ROS production compared to other treatments (Figure 8). ROS is produced mainly by the mitochondria in diabetes [75]. The nanoparticle-mediated efficient intracellular delivery can enhance the curcumin’s ROS inhibitory activity [76], which was confirmed by our results using the curcumin-loaded SEDDS.

### 3.9. Cellular Uptake

**Figure 9:**
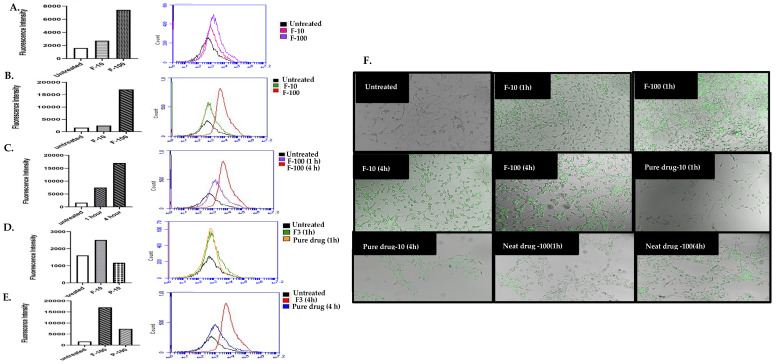
Cellular uptake of the curcumin-loaded SEDDS (F3 formulation) in NIH3T3 fibroblasts, determined by FACS. (**A**) one-hour cell incubation at different doses of F3 SEDDS; (**B**) four-hour cell incubation at different doses of F3 SEDDS; (**C**) time-dependent uptake of F3 SEDDS at 100 µg/mL curcumin dose; (**D**) F3 SEDDS vs. pure drug at 10 µg/mL curcumin after 1 h cell incubation; (**E**), F3 SEDDS vs. pure drug at 100 µg/mL after 4 h cell incubation; and (**F**) fluorescence microscopy images. F10: F3 SEDDS at 10 µg/mL curcumin. F100: F3 SEDDS at 100 µg/mL curcumin.

The flow cytometry data suggested that the F3 SEDDS formulation enhanced the cellular uptake of curcumin compared to the pure drug in NIH3T3 cells (Figure 9D). In addition, the cellular uptake of the F3 SEDDS was dose- and time-dependent (Figure 9A–E). It is known that the nanocarriers are internalized mainly via the efficient endocytic pathways [77], while pure drug internalization is particularly diffusion dependent. The poorly water-soluble curcumin makes drug administration difficult, further reducing its internalization and resulting in lowered therapeutic effects [78,79]. 

The increased cellular uptake of the curcumin-loaded SEDDS enhanced curcumin’s antioxidant effects (Figure 8) and facilitated the fibroblast migration (Figure 7). A linear dependency on the uptake of SEDDS by the fibroblasts was observed, which was found to be dose-dependent. The cellular uptake of curcumin is facilitated when it is formulated into nanocarriers with the most negligible cytotoxicity compared to free drug solution [80,81,82,83].

### 3.10. Diabetic Wound Healing

**Figure 10:**
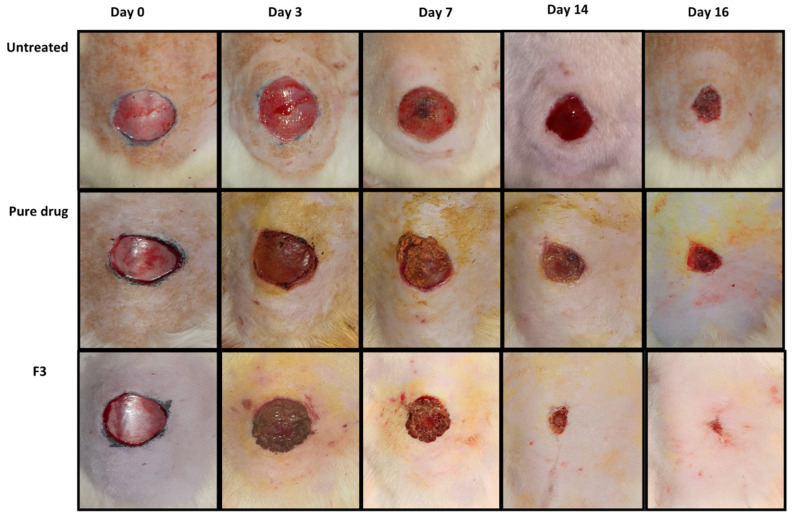
Photos of skin wounds upon treatment in the diabetic rats.

**Figure 11:**
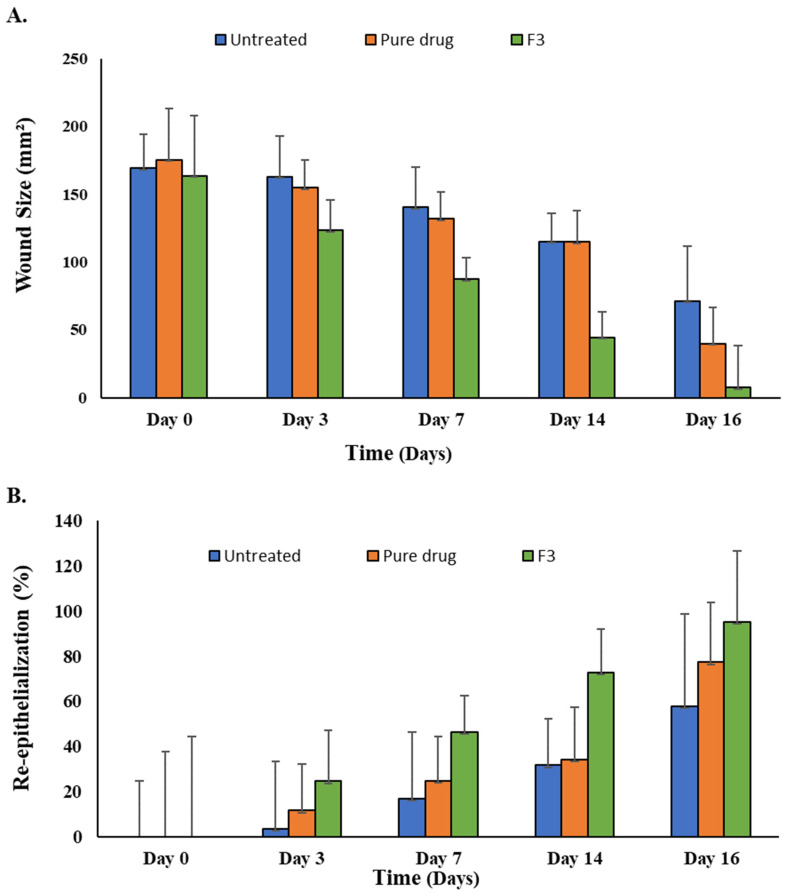
(**A**) Wound sizes (mm^2^) and (**B**) percentage re-epithelialization in the diabetic rats upon treatment.

The in vivo wound healing capability of the SEDDS formulation (F3) was evaluated in the diabetic rat model. The photos of the skin wounds are shown in Figure 10, and the wound size and re-epithelialization results are shown in Figure 11. The curcumin-loaded SEDDS-treated diabetic wound exhibited an accelerated wound healing process and hastened the skin re-epithelialization process compared to the untreated, blank formulation-treated, or pure drug-treated animals. On day 16, the F3 formulation rats showed almost complete wound healing compared to the pure drug-treated group (77%) and untreated group (58%).

The data suggested that curcumin’s therapeutic effects on wound healing and skin tissue regeneration were significantly improved while curcumin’s toxicity was minimized when the curcumin was formulated into the SEDDS. Using the free form of curcumin by adding it in DMSO and PBS 7.4 initiated precipitation and accumulation on the skin surface, which is not a favorable phenomenon in dermal delivery [64].

The in vivo results are well consistent with the in vitro cytotoxicity, cellular uptake, ROS inhibition, and cell migration data. Here, the SEDDS formulation increased the drug solubility, decreased the drug degradation, slowed down the drug release, and increased the drug uptake and efficacy at the wound site resulting in improved, persistent therapeutic effects to accelerate the wound healing process in the diabetic rats [32,36]. Curcumin promoted the migration of epithelial cells at the skin surface to the wound site in the proliferative stage, which was evident in the percentage re-epithelialization results (Figure 11B), which were significantly higher (ANOVA, *p* < 0.05) in the case when SEDDS were applied. 

In wound healing, increased division and migration of epithelial cells along with keratinocytes occur from the periphery towards the wound site, both of which depend upon the interaction of keratinocytes with the extracellular matrix at the wound surface [84]. Curcumin promotes wound healing owing to its multiple therapeutic effects, i.e., antioxidant and anti-inflammatory properties [8], increased fibronectin production, collagen deposition, and myofibroblast contraction [85], thereby, increasing the expression of anti-inflammatory cytokines (IL-10), reducing the levels of inflammatory cytokines (matrix metalloproteinase-9, TNF-α, and IL-1β), increasing antioxidant enzymes (glutathione peroxidase, superoxide dismutase, and catalase), downregulating the NF-kB signaling pathway, and enhancing neovascularization [8,86].

## 4. Conclusions

This study aimed at developing a curcumin-loaded self-emulsifying drug delivery system with increased drug solubility, stability, sustained drug release, lowered cytotoxicity, increased cellular uptake, and improved therapeutic effects. The results indicated that curcumin in SEDDS is superior in exerting its wound healing activity compared to the pure drug in solution form. The improved wound contraction by curcumin SEDDS was possible due to nano-droplets of the formulation, which enhanced the drug efficacy as a healing agent. This was evident from the higher cellular uptake of drug by fibroblast cells. 

Curcumin SEDDS accelerated the healing process by reducing inflammation along with early proliferation, collagen formation, and epithelialization through regulating the expression of certain pro-inflammatory cytokines and growth factors responsible for delaying diabetic wound healing. In summary, curcumin-loaded SEDDS may have great potential in treating diabetic wounds and accelerating skin tissue regeneration.

## Figures and Tables

**Table 1:** The results of formulation optimization of curcumin-loaded SEDDS.

Formulations	Drug (mg)	Tween 80 (mg)	PEG 600 (mg)	Ethyl Oleate (mg)	Water (mg)	Observation	Grade	Emulsification Time (Min)
**F1**	4	83	143	120	650	Stable with no sedimentation/creaming	A	<1
**F2**	4	87	148	111	650	Stable with no sedimentation/creaming	A	<1
**F3**	4	90	156	100	650	Stable with no sedimentation/creaming	A	<1
**F4**	4	100	160	136	600	Creaming at the surface. No drug settling	B	2
**F5**	4	116	160	120	600	Creaming at the surface. No drug settling	B	2
**F6**	4	126	160	110	600	Creaming at the surface. No drug settling	B	2
**F7**	4	136	160	100	600	Creaming at the surface. No drug settling	B	2
**F8**	4	105	166	135	590	Creaming at the surface. No drug settling	B	2
**F9**	4	108	169	139	580	Creaming at the surface. No drug settling	B	2
**F10**	4	111	172	143	570	Creaming at the surface. No drug settling	B	2
**F11**	4	114	175	147	560	Creaming at the surface. Little Drug settling	C	3
**F12**	4	117	178	151	550	Creaming at the surface. No drug settling	B	2
**F13**	4	120	181	155	540	Creaming at the surface. No drug settling	B	2
**F14**	4	123	184	159	530	Creaming at the surface. No drug settling	B	2
**F15**	4	126	187	163	520	Creaming at the surface. No drug settling	B	2
**F16**	4	129	190	167	510	Creaming at the surface. No drug settling	B	2
**F17**	4	132	193	171	500	Creaming at the surface. No drug settling	B	2
**F18**	4	135	196	175	490	Creaming at the surface. No drug settling	B	2
**F19**	4	138	199	179	480	Creaming at the surface. No drug settling	B	2
**F20**	4	72	133	91	700	Creaming at the surface. Little drug settling	C	3
**F21**	4	57	118	71	750	Creaming at the surface. Little drug settling	C	3
**F22**	4	42	103	51	800	Creaming at the surface. Little drug settling	C	3

**Note.** Grade A: rapidly forms emulsions with a clear appearance; Grade B: rapidly forms emulsions with a slightly less clarity and a whitish appearance; Grade C: forms milky emulsions; Grade D: slow in emulsification and results in a dull, greyish white emulsion with a light oily appearance; and Grade E: difficult in emulsification and with large oil droplets or phase separation.

**Table 2:** Thermodynamic stability of the curcumin-loaded SEDDS.

Formulations	Grade	Thermodynamic Stability Tests			
		Centrifugation	Heating and Cooling Cycle	Freeze–Thaw Cycle	Robustness to Dilution
**F1**	A	Pass	Pass	Pass	Pass
**F2**	A	Pass	Pass	Pass	Pass
**F3**	A	Pass	Pass	Pass	Pass

## Data Availability

Data is contained within the article.
